# The Effect of Vibration Characteristics on the Atomization Rate in a Micro-Tapered Aperture Atomizer

**DOI:** 10.3390/s18040934

**Published:** 2018-03-21

**Authors:** Qiufeng Yan, Jianhui Zhang, Jun Huang, Ying Wang

**Affiliations:** 1 State Key Laboratory of Mechanics and Control of Mechanical Structures, Nanjing University of Aeronautics and Astronautics, Nanjing 210016, China; kangy@nuaa.edu.cn; 2 College of Mechanical and Electrical Engineering, Guangzhou University, Guanzhou 510006, China; 3 Research Center of Fluid Machinery Engineering and Technology, Jiangsu University, Zhenjiang 212013, China; 4 Taizhou Polytechnical Institute, Taizhou 225300, China; wangying@tzpc.edu.cn

**Keywords:** micro-tapered aperture, angle changes, atomization rate, vibration characteristics

## Abstract

Because little is known about the atomization theory of a micro-tapered aperture atomizer, we investigated the vibration characteristics of this type of atomizer. The atomization mechanism of a micro-tapered aperture atomizer was described, and the atomization rate equation was deduced. As observed via microscopy, the angle of the micro-tapered aperture changes with the applied voltage, which proved the existence of a dynamic cone angle. The forward and reverse atomization rates were measured at various voltages, and the influence of the micro-tapered aperture and its variation on the atomization rate was characterized. The resonance frequency of the piezoelectric vibrator was obtained using a laser vibrometer, and the atomization rates were measured at each resonance frequency. From experiments, we found that the atomization rates at the first five resonance frequencies increased as the working frequency increased. At the fifth resonance frequency (121.1 kHz), the atomization rate was maximized (0.561 mL/min), and at the sixth resonance frequency (148.3 kHz), the atomization rate decreased significantly (0.198 mL/min). The experimental results show that the vibration characteristics of the piezoelectric vibrator have a relatively strong impact on the atomization rate. This research is expected to contribute to the manufacture of micro-tapered aperture atomizers.

## 1. Introduction

Traditional atomization and ejection devices driven by pneumatic air or ultrasound are widely used in various processes, including inhalation therapy [1,2,3], spray drying [4], mass spectrometry [5], printed circuits [6], 3D prototyping [7], and precise surface coating [8,9]. The development of finer droplets and smaller devices has emerged as the latest trend in atomization and ejection technology, as illustrated by the ultrasonic atomizers developed by Yabe et al. [10] and Fu et al. [11]. Surface acoustic wave atomizers release energy into the liquid system to break the surface tension of the liquid and allow the droplets to escape from the surface for atomization and ejection. Droplets are formed only at the surface, but the energy is applied to the entire liquid system. Therefore, breaking the liquid into droplets and ejecting the resulting particles in this manner is neither cost-effective nor energy-efficient. In addition, the droplets are formed and ejected in a random and uncontrolled manner, producing a wide range of droplet sizes.

To improve energy use and minimize the range of droplet sizes, atomizers with micro-tapered aperture dispensers that are actuated by high-frequency piezoceramic rings have been developed. In 1986, Maehara et al. proposed an atomizer with this structure and improved its structure [12]. Maehara et al. found that the atomization rate at the second-order resonance frequency is larger than that at the first-order resonance frequency [12,13]. Experiments performed by Shen et al. [14,15] showed that the problems caused by low energy use, large droplet size, and a wide droplet size range can be eliminated when micro-tapered apertures are used. This unit has contributed significantly to the atomizer industry because of its mobility and miniaturization, which represent revolutionary changes in the inhalation therapy field. 

A substantial amount of research has been conducted on micro-aperture atomizers; the present study focuses on the atomizer’s structure and performance, and the principle of atomization is not a focus of this study. Lu et al. suggested that the dispenser pumping mechanism depends on the balance between the inertial and capillary forces [16,17]. They also explained how this type of atomizer works: liquid is pinched off as droplets pass through the cone nozzle during forward vibration and then adheres to the orifice through capillary force during backward vibration. Driven by the high-frequency piezoelectric ceramic ring, the liquid is repeatedly pinched off and attached, resulting in atomization. 

The concept of the dynamic cone angle was proposed in our previous research [18], but the pumping mechanism has not been analyzed. In this study, using the vibration characteristics of the piezoelectric vibrator as the entry point, the atomization mechanism of the micro-tapered aperture atomizer was analyzed, and the calculation equation of atomization was derived. We found that changes in liquid and cone volumes, operating frequency, and flow resistance affect the atomization rate. Microscopy showed that the angle of the micro-tapered aperture changes with voltage. The forward and reverse atomization rates were measured at various voltages. In addition, the influence of the micro-tapered hole and its variation on the atomization rate was characterized. Finally, the atomization rate at various resonance frequencies was measured. The experimental results show that the vibration characteristics of piezoelectric vibrators have a relatively strong impact on the amount of atomization.

## 2. Principle of Atomization

**Figure 1:**
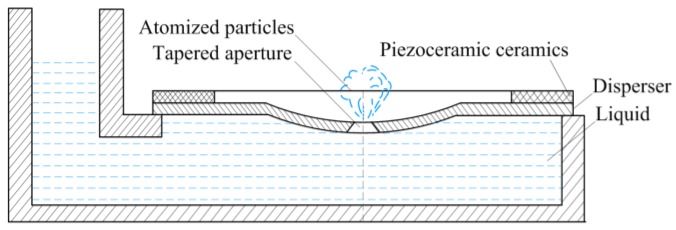
Illustration of the principle of atomization.

The fluid in the micro-tapered aperture exists in two environments: one is external, where the fluid is united with the atmosphere, and the other is the liquid chamber. During operation, the small side of the cone aperture contacts the atmosphere, and the large side of the cone aperture contacts the liquid, as shown in Figure 1.

The deformation process of the dispenser is both small and periodic. In this cycle, a neutral surface with no strain or stress exists. The metal sheets at either side of this neutral surface are either stretched or compressed. 

**Figure 2:**
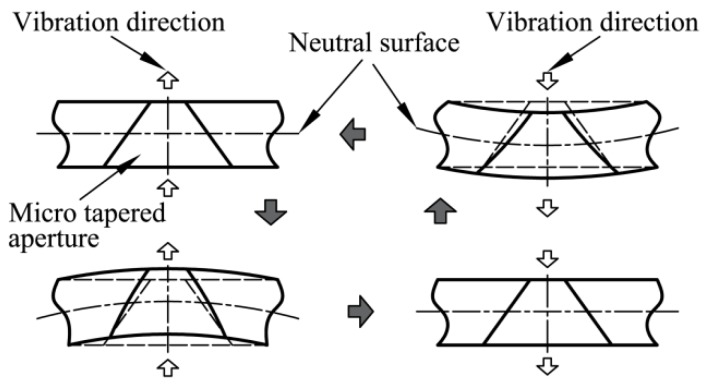
Dynamic cone angle developed by deformation of the micro-tapered aperture.

When driven by periodic stimulated vibrations, a point on the non-neutral surface moves in two possible cycles: (1) the point is stretched from equilibrium to its limit, released back to equilibrium, compressed from equilibrium to its limit, and then released back to equilibrium; or (2) the point is compressed from equilibrium to its limit, released back to equilibrium, stretched from equilibrium to its limit, and then released back to equilibrium. Figure 2 shows how the micro-tapered aperture changes in a single cycle. 

When the volume on the upper side decreases, the cone angle and pressure increase. The high pressure induces flow towards the region of low pressure. One branch flows to the lower side to occupy the additional space caused by the large aperture volume, while the other branch is pushed towards the exterior with lower pressure. The low pressure at the lower side causes the liquid to flow into that side, where the liquid accumulates from the upper side and the liquid chamber. When the volume on the upper side increases, the cone angle decreases. This effect reduces the pressure and results in a flow into the upper side. One branch consists of the flow that was once moving towards the exterior, and the other branch flows in from the lower side. On the lower side, higher pressures cause the flow to move outwards to the upper side and to the liquid chamber.

## 3. Theoretical Calculations

### 3.1. Volume Change of the Liquid Chamber 

Based on the atomization principle described above, pressure atomization is caused by the volume change in the liquid chamber, which is caused by the vibration of the piezoelectric vibrator. To simplify the calculation, the following conditions are assumed: (1) when the dispenser is vibrating in the upward and downward directions, the deformation and velocity are symmetrical with respect to the original neutral surface; and (2) the dispenser vibrations satisfy the Kirchhoff hypothesis.

We assume that the piezoelectric vibrator radius is *R* and that the maximum amplitude is *w*_0_. A polar coordinate system is established on the piezoelectric vibrator, where the origin of the coordinate is the center of the piezoelectric vibrator, the polar axis is along the radius direction, and the axis of ordinate is along the vibration direction of the piezoelectric vibrator. When the piezoelectric vibrator moves to the limit position, the parabolic equation of the piezoelectric vibrator is as follows [18]:

(1) $$w(r)=w_{0}(1-\frac{r^{2}}{R^{2}})$$

where *w*(*r*) is the parabolic equation of the piezoelectric vibrator.

When the piezoelectric vibrator moves from the equilibrium position to the extreme position, the volume of the liquid chamber changes is as follows:

(2) $$\Delta V_{VS}=2\pi \int_{0}^{R}w_{0}(1-\frac{r^{2}}{R^{2}})rdr=\frac{\pi w_{0}R^{2}}{2}$$

where Δ*V_VS_* is the volume of the liquid chamber changes.

### 3.2. Volume Change of the Cone Apertures 

**Figure 3:**
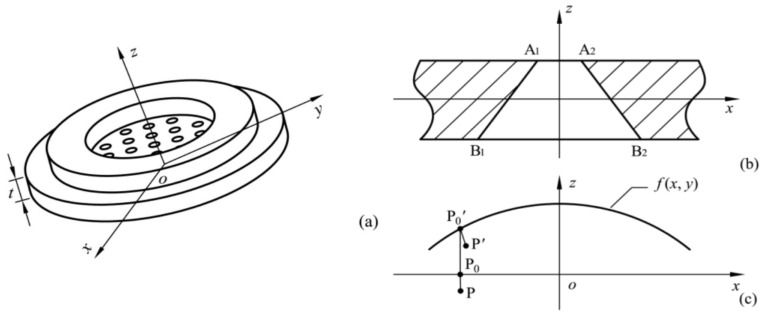
Schematic coordinate of the dispenser. (**a**) Coordinate system of the dispenser; (**b**) Profile of a tapered aperture; (**c**) Deformation process of a point in the dispenser.

As mentioned in a previous report [19], the neutral surface of the dispenser is the *x*–*y* plane, and the *z*-axis is perpendicular to this plane, as shown in Figure 3a. To clearly illustrate the deformation of the apertures, the *x*–*z* plane is shown in Figure 3b. For a neutral surface of *z* = *f*(*x*, *y*) after deformation, the movement from P to P′ on the dispenser is shown in Figure 3c.

The volume of the cone apertures changes is as follows [19]:

(3) $$\Delta V_{VD}=\underset{\Omega }{∭}[2z(f_{yy}+f_{xx}+f_{y}^{2}f_{xx}-2f_{x}f_{y}f_{xy}+f_{x}^{2}f_{yy})m^{-1}]dV$$

where Δ*V_VD_* is the volume of the cone apertures changes.

### 3.3. Flow Resistance Analysis

In practice, the geometry of the micro-tapered aperture changes with the periodic changes of the metal substrate; the angle of the micro-aperture constantly changes, changing the forward and reverse flow resistance. Therefore, the average cyclic resistance is discussed below.

The average forward and reverse flow resistances of the cone apertures are as follows:

(4) $${\bar{\xi (\chi )}}_{+}=\frac{\int_{\chi \to \infty }\xi {\left(\chi \right)}_{+}d\chi }{\chi }$$

(5) $${\bar{\xi (\chi )}}_{-}=\frac{\int_{\chi \to \infty }\xi {\left(\chi \right)}_{-}d\chi }{\chi }$$

where $\xi {\left(\chi \right)}_{+}$ and $\xi {\left(\chi \right)}_{-}$ are the instantaneous flow resistance in the forward and reverse directions, respectively. $\bar{\xi {\left(\chi \right)}_{+}}$ and $\bar{\xi {\left(\chi \right)}_{-}}$ are the average forward and reverse flow resistances of the cone apertures, respectively.

We discuss only the case in which the angle of the micro-aperture is greater than 40°. Previous studies [20] have indicated that when the angle of the micro-tapered aperture is greater than 30°, the flow resistance of the gradually expanding flow is greater than that of the tapered flow. Therefore, the flow through the tapered tube is greater than that of the gradually expanding tube under the same conditions.

The flow resistance of fluid flow through the tapered bore into the external environment is defined as follows:

(6) $$\xi_{v}=\frac{\bar{\xi {\left(\chi \right)}_{+}}-\bar{\xi {\left(\chi \right)}_{-}}}{2+\bar{\xi {\left(\chi \right)}_{+}}+\bar{\xi {\left(\chi \right)}_{-}}}$$

where *ξ_v_* is the flow resistance of fluid flow through the tapered bore into the external environment.

### 3.4. Atomization Rate

Due to the difference between the diffuser flow resistance and the nozzle flow resistance, one-way flow is produced by the volume changes in the micro-tapered aperture and the liquid chamber. Olsson [21] compared the results obtained from the numerical calculation for conical flows and those mentioned in [22] and found that the flow resistances in the nozzle/diffuser elements at the micro-scale are similar to those at the macro-scale. Therefore, the atomization process of the atomizer can be regarded as the working process of a valveless piezoelectric pump. We can find the flow equation of the valveless piezoelectric pump in [20]. Atomization is caused by the vibrator volume change and micro-tapered aperture volume product change acting in conjunction. Therefore, by the flow equation of the valveless piezoelectric pump, the atomization rate can be calculated using Equation (7):

(7) $$Q=\left(\Delta V_{VS}+n\Delta V_{VD}\right)f\xi_{v}$$

where Δ*V_VS_* is the change in the liquid chamber volume, Δ*V_VD_* is the volume change in the micro-aperture, *ƒ* is the working frequency, n is the number of apertures, and *ξ_v_* can be obtained from Equation (6).

From the equation of atomization, we find that changes in the liquid and cone volumes, the operating frequency, and the flow resistance affect the atomization rate. The working frequency also affects the vibration speed and amplitude of the piezoelectric vibrator. Furthermore, the change in the amplitude directly affects the volume of the liquid chamber, and the micro-aperture and affects the flow resistance, thereby further affecting the atomization rate. The vibration characteristics of the piezoelectric vibrator affect the pumping ability of the valveless piezoelectric pump; therefore, these characteristics also affect the atomization rate of the micro-aperture atomizer.

## 4. Design of Experiments

**Figure 4:**
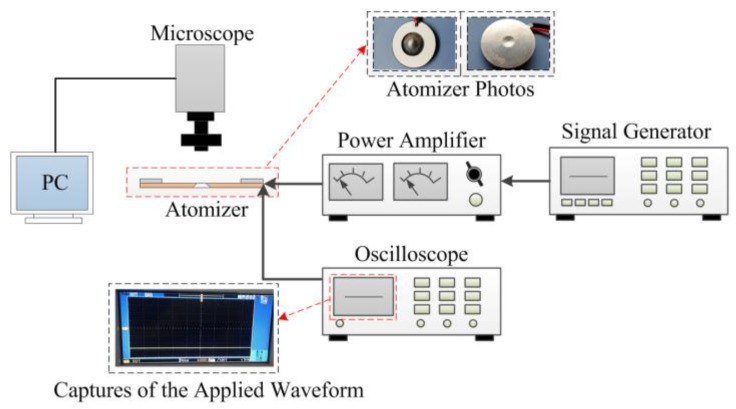
Method used to measure the micro-aperture and the captures of the applied waveform.

Table 1 shows the parameters of the atomizer used in this study. The diameter of the micro-aperture was measured using the method shown in Figure 4, and the other parameters were measured using a Vernier caliper.

Figure 4 shows the method used to measure the micro-tapered aperture, and the captures of the applied waveform. Under an AC voltage, the geometry of the micro-tapered aperture on the dispenser changes as the dispenser vibrates. However, it is not possible to directly measure the size of the micro-tapered aperture. In this experiment, we adjusted the signal generator to output DC voltage and used the power amplifier to amplify the DC voltage and then deliver it to the atomizer. The DC voltage was monitored by the oscilloscope. The atomizer obtained different DC voltages from the power amplifier. The size of the micro-tapered aperture under different voltages was measured with a microscope to obtain the relationship between the diameter of the tapered hole and the voltage. 

**Figure 5:**
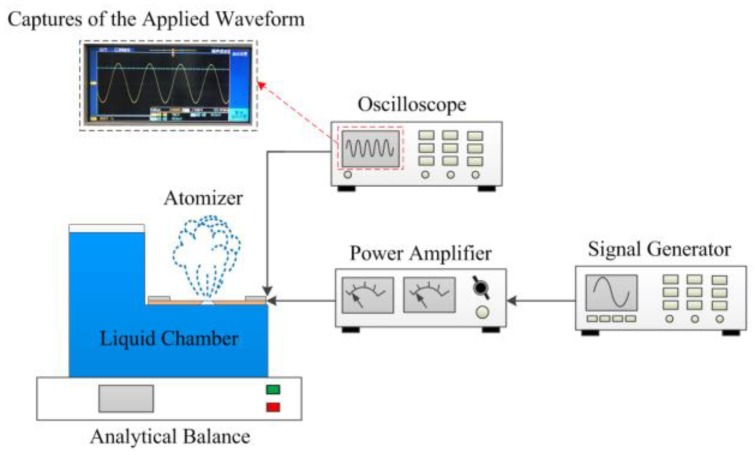
Measurement of the atomization rate and the captures of the applied waveform.

Figure 5 shows the measurement of the atomization rate and the captures of the applied waveform. In this experiment, we adjusted the signal generator to output AC voltage and used the power amplifier to amplify the AC voltage and then deliver it to the atomizer. The AC voltage was monitored by the oscilloscope. While the atomization rate was being measured, the atomizer was placed on a high-precision analytical balance, and a stopwatch was used to time the power supply to the atomizer. We stopped supplying the voltage to the atomizer after 1 min, and the atomization rate was obtained by measuring the reduction of liquid in the liquid chamber per minute using a high-precision analytical balance. To verify the influence of the micro-tapered aperture and its variation on the atomization rate, the piezoelectric ceramic was attached to the positive and negative surfaces of the substrate with the conical aperture to obtain the forward-direction and reverse-direction atomization rates, respectively.

**Figure 6:**
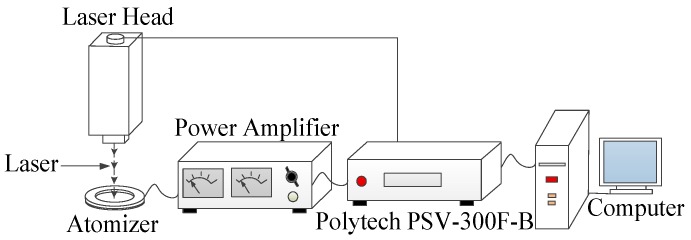
The method used to measure the vibration mode of the piezoelectric vibrator.

Figure 6 shows the method of measuring the vibration mode of the piezoelectric vibrator. The velocity and amplitude curves of the atomized tablets were obtained using a Polytech PSV-300F-B laser vibrometer. The resonance frequency was obtained from the curve, and the resonance point was determined. In addition, the atomization rate was measured at each resonance point at an operating voltage of 70 V.

## 5. Results and Discussion

**Figure 7:**
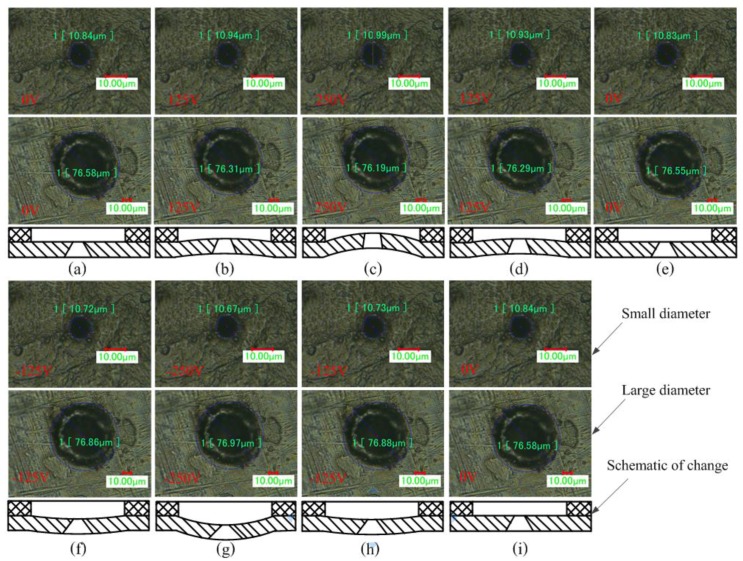
Measurement results of the aperture parameters and corresponding schematics. (**a**) First measurement, applied DC voltage of 0 V; (**b**) second measurement, applied DC voltage of 125 V; (**c**) third measurement, applied DC voltage of 250 V; (**d**) fourth measurement, applied DC voltage of 125 V; (**e**) fifth measurement, applied DC voltage of 0 V; (**f**) sixth measurement, applied DC voltage of −125 V; (**g**) seventh measurement, applied DC voltage of −250 V; (**h**) eighth measurement, applied DC voltage of −125 V; (**i**) ninth measurement, applied DC voltage of 0 V.

Figure 7 shows the results of measuring the micro-tapered aperture at various voltages along with schematics of the change. Figure 7a–i show the results of nine measurements at various voltages and a schematic of the change in the micro-tapered aperture. Figure 7 shows that the geometry of the micro-tapered aperture changes under various voltages. In this experiment, the voltage applied is a DC voltage; however, in the actual process, the voltage applied to the atomizer is an AC voltage. The change in the geometry of the micro-tapered aperture is more pronounced in the actual process. The experiment shows the change in the micro-tapered aperture and establishes the concept of the dynamic cone angle proposed in our previous research results.

Table 2 shows the results of measuring the micro-tapered aperture at different voltages. Under different voltages, the tapered-side diameter of the aperture, the flared-side diameter of the aperture, and the angle of the aperture all changed.

**Figure 8:**
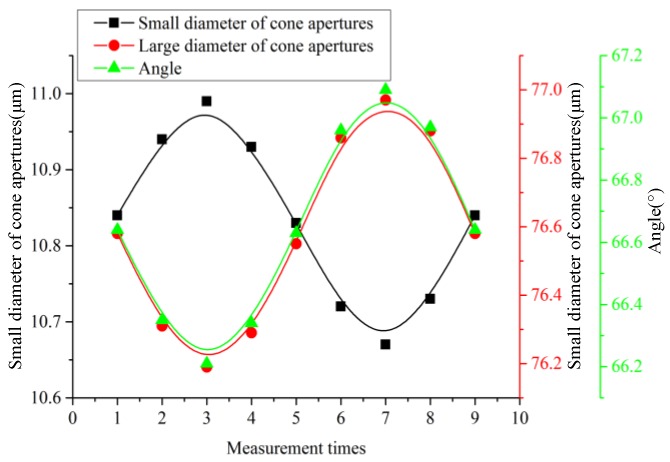
Variation in the parameters of the micro-tapered aperture with applied voltage.

Based on the values listed in Table 2, the relationship of the tapered-side diameter of the aperture, the flared-side diameter of the aperture, the angle of aperture, and the driving voltage are shown in Figure 8. The three parameters vary sinusoidally, with the tapered-side diameter of the aperture exhibiting a positive sinusoid and the flared-side diameter and the angle of the aperture varying in the opposite direction.

**Figure 9:**
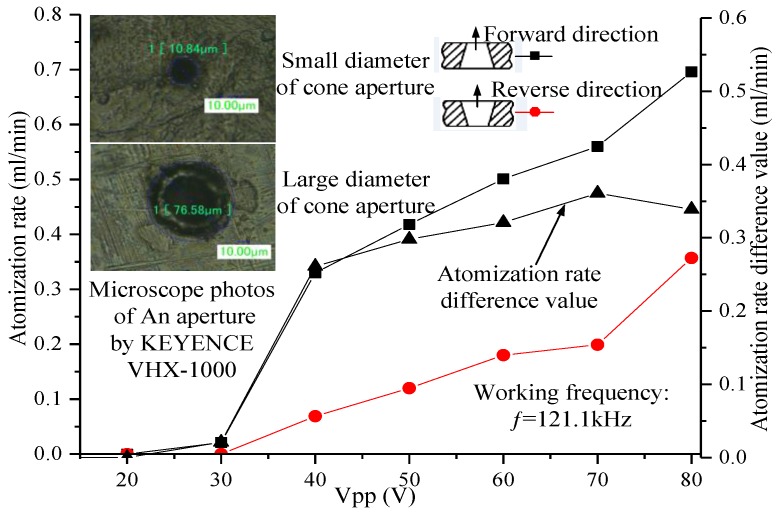
Variation in the atomization rate and the atomization rate difference values with applied voltage when the micro-tapered aperture atomizer is operating in the forward and reverse directions.

The atomization rates were measured at both the flared (forward-direction) and tapered (reverse-direction) sides under different applied voltages at 121.1 kHz, and the difference values were calculated and are plotted in Figure 9. No visible atomization was observed when the voltage was lower than 30 V; instead, drops were observed only at the micro-tapered aperture in both the forward and reverse directions. For the micro-tapered aperture operating in the forward direction, visible atomization was generated when the applied voltage was higher than 30 V, and the atomization rate increased when the applied voltage increased. For the micro-tapered aperture operating in the reverse direction, only water drops were formed even when the voltage was as high as 70 V. However, when the voltage reached 80 V, atomization began. The atomization rate measured in the forward direction was much higher than that measured in the reverse direction. This atomization rate difference increased as the applied voltage increased.

In the test described above, the atomization rate measured in the forward direction is much higher than that measured in the reverse direction, which indicates that the micro-tapered aperture plays an important role in the atomization process. When the liquid is located in the flared side of the micro-tapered aperture, the atomization rate is equal to the sum of the atomization rate caused by the change in the volume of the piezoelectric vibrator and the atomization rate caused by the change in the volume of the micro-tapered aperture. When the liquid is located on the tapered side of the micro-tapered aperture, the pumping effect caused by the change in the volume of the aperture partially atomizes liquid into the liquid chamber. The atomization rate is equal to the atomization rate caused by the change in the volume of the piezoelectric vibrator minus the atomization rate caused by the change in the volume of the micro-tapered aperture. Therefore, the atomization rate in the forward direction is far greater than that in the reverse direction. With increasing voltage, the deformation of the piezoelectric vibrator increases, and the volume change in the piezoelectric vibrator and micro-tapered aperture increases. Thus, the angle of the micro-tapered aperture and *ξ_v_* in Equation (6) also increase. From Equation (7), the atomization rate is shown to increase with increasing voltage.

**Figure 10:**
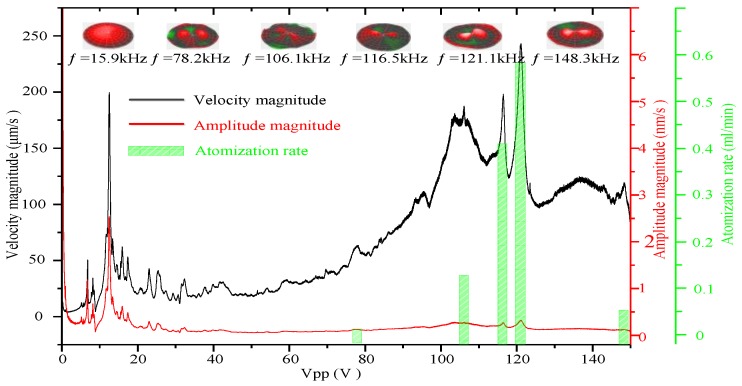
Frequency sweep curves of the vibration velocity and amplitude, vibration modes of resonance points, and atomization rates at the resonance points.

Figure 10 shows the frequency sweep curves of vibration velocity and vibration amplitude, vibration modes of resonance points, and atomization rates at the resonance points. Table 3 shows the resonant frequencies and the atomization rates of each resonant frequencies under an applied voltage of 70 V. In this experiment, no visible atomization was observed below a resonant frequency of 15.9 kHz. The atomization rates increased gradually with resonance frequency, and at the resonance frequency of 121.1 kHz, the atomization rate was maximized (0.561 mL/min). However, at the resonance frequency of 148.3 kHz, the atomization rate decreased significantly.

Equation (7) indicates that the working frequency, the volume change in the piezoelectric vibrator, and the volume change in the micro-tapered aperture affect the atomization rate. The vibration amplitude of the piezoelectric vibrator determines the volume changes of the piezoelectric vibrator and the micro-tapered aperture. In addition, the volume change increases with the vibration amplitude. As shown in Figure 10, the vibration amplitude is highest at the first resonance frequency (15.9 kHz); however, this condition does not cause atomization due to the lower operating frequency. From the second to the fifth resonance frequency, the vibration amplitude gradually increases, and the volume changes in the piezoelectric vibrator and micro-tapered aperture also increase. Additionally, the operating frequency also increases, raising the atomization rate. At the sixth resonance frequency (148.3 kHz), which is higher than the fifth resonance frequency (121.1 kHz), the vibration amplitude of the piezoelectric vibrator is much smaller than the vibration amplitude in the fifth resonance frequency. Equation (7) indicates that the atomization rate decreases significantly.

According to the working principle of the atomizer proposed by Lu, the atomization rate of the forward direction should be consistent with that of the reverse direction; however, the atomization rate measured in the forward direction was much higher than that measured in the reverse direction, as shown in Figure 9. The atomization rate was also expected to increase as the operating frequency increases; for example, at the resonance frequency of 148.3 kHz, the atomization rate was expected to be greater than the atomization rate at the resonance frequency of 121.1 kHz. A substantial reduction in the atomization rate was observed as the operating frequency increased. However, the working principle of the atomizer examined in this study and the manner in which the vibration characteristics of the piezoelectric vibrator affect the pumping effect can explain these phenomena.

## 6. Conclusions

By investigating the vibration characteristics of the piezoelectric vibrator, the micro-tapered aperture atomizer’s atomization mechanism was analyzed, and its equation of atomization was derived. From the equation of atomization, we found that changes in the liquid and cone volumes, operating frequency, and flow resistance affect the atomization rate. As observed via microscopy, the angle of the micro-tapered aperture changes with the applied voltage. We measured the atomization rates of the forward and reverse directions and found that they increased as the applied voltage increased. The atomization rate of the forward direction was also found to be much higher than that of the reverse direction. The influence of the micro-tapered aperture and its variation on the atomization rate was demonstrated. A comparison of the atomization rates of the six resonance frequencies showed that the atomization rates at the first five resonance frequencies increased as the working frequency increased. At the fifth resonance frequency (121.1 kHz), the atomization rate was maximized (0.561 mL/min). At the sixth resonance frequency (148.3 kHz), the atomization rate decreased significantly (0.198 mL/min). The cause of this decrease was the reduction in the vibration amplitude of the piezoelectric vibrator, which decreased the change in volume in the liquid chamber and the micro-tapered aperture, weakening the pumping effect of the micro-tapered aperture.

## Figures and Tables

**Table 1:** Geometric parameters of the atomizer.

Geometric Parameters of the Atomizer	
Outer diameter of piezoelectric ceramic ring (mm)	15.96
Inner diameter of piezoelectric ceramic ring (mm)	7.69
Thickness of piezoelectric ceramic ring (mm)	0.63
Diameter of the dispenser (mm)	15.96
Thickness of the dispenser (μm)	50
Large diameter of cone apertures (μm)	76.58
Small diameter of cone apertures (μm)	10.84

**Table 2:** Parameters of the micro-tapered aperture at different voltages.

Measurement Time	U (V)	*d*_1_ (μm)	*d*_2_ (μm)	*t* (μm)	Angle (°)
1	0	10.84	76.58	50	66.64
2	150	10.94	76.31	50	66.35
3	300	10.99	76.19	50	66.21
4	150	10.93	76.29	50	66.34
5	0	10.83	76.55	50	66.63
6	−150	10.72	76.86	50	66.96
7	−300	10.67	76.97	50	67.09
8	−150	10.73	76.88	50	66.97
9	0	10.84	76.58	50	66.64

**Table 3:** Resonant frequencies and atomization rates of each resonant frequencies under an applied voltage of 70 V.

Frequency (kHz)	Atomization Rate (mL/min)
15.9	-
78.2	0.019
106.1	0.133
116.5	0.411
121.1	0.561
148.3	0.198
